# The effects of daily meteorological perturbation on pregnancy outcome: follow-up of a cohort of young women undergoing IVF treatment

**DOI:** 10.1186/s12940-019-0538-7

**Published:** 2019-11-28

**Authors:** Mingpeng Zhao, Haoyang Zhang, Tarah H. B. Waters, Jacqueline Pui Wah Chung, Tin Chiu Li, David Yiu Leung Chan

**Affiliations:** 1 Assisted Reproductive Technology Unit, Department of Obstetrics and Gynaecology, Faculty of Medicine, The Chinese University of Hong Kong, Block E, 9F, Special Block, Prince of Wales Hospital, 30-32 Ngan Shing Street, Hong Kong, China; 2 School of Data and Computer Science, Sun Yat-sen University, Guangzhou, China

**Keywords:** Seasonality, Temperature, Pregnancy rate, IVF, Long ovarian protocol

## Abstract

**Background:**

Human reproduction follows a seasonal pattern with respect to spontaneous conception, a phenomenon wherein the effect of meteorological fluctuations might not be unique. However, the effect of seasonal variations on patients who underwent in vitro fertilization (IVF) treatment is unclear. We aimed to evaluate the effects of meteorological variation on the pregnancy rate in a cohort undergoing IVF treatment by performing multivariable analyses.

**Methods:**

We conducted a cohort study in a sub-tropical region with prominent seasonal variations (2005–2016). Women aged < 35 years who were treated with a long ovarian stimulation protocol and underwent fresh embryo transfer (ER) were included. Data on gonadotropin administration (CYCL), oocyte retrieval (OR), ER, and pregnancy outcomes were prospectively recorded. For each patient, the daily average of meteorological data (temperature, humidity, sunlight duration, solar radiation) was recorded from the date of CYCL to ER. Multiple logistic regression analysis adjusted for age, fertilization method, year of the cycle, gonadotropin dose, and transferred embryo grade was performed to determine the relationship between the meteorological parameters and clinical pregnancy. Patients with one successful cycle and one failed cycle were subtracted for a case-control subgroup analysis through mixed effect logistics regressions. Time-series analysis of data in the epidemic level was conducted using the distributed lag linear and non-linear models (DLNMs).

**Results:**

There were 1029 fresh cycles in 860 women (mean age 31.9 ± 2.0 years). Higher mean temperature from CYCL to OR (adjusted odds ratio [aOR] 1.04; 95% confidence interval [CI] 1.01–1.07, *P* = 0.01) increased the odds of pregnancy, while OR to ER did not show any statistical significance. Compared to that in winter, the odds of becoming pregnant were higher during higher temperature seasons, summer and autumn (aOR 1.47, 95%CI 0.97–2.23, *P* = 0.07 (marginally significant) and aOR 1.73, 95%CI 1.12–2.68, *P* = 0.02, respectively). Humidity, sunlight duration, and solar radiation had no effect on the outcome. The subgroup analysis confirmed this finding. The time-series analysis revealed a positive association between temperature and relative risk for pregnancy.

**Conclusions:**

In IVF treatment, the ambient temperature variation alters the pregnancy rates; this aspect must be considered when obtaining patient consent for assisted conception.

## Background

Seasonal changes in human reproduction have been observed in many studies [1–3]. There is a seasonal pattern in the rate of natural conception in women [4–6]. In warm areas, a peak in natural conception was observed during winter [7], whereas, in cold areas, the peak was observed during summer [8]. The seasonal patterns in human reproduction are consistent with the meteorological change. However, the sole effect of the meteorological change on human reproduction may be masked by other factors, such as food availability, seasonal movement, social-cultural factors, and other such factors [2].

Assisted reproduction provides a good model for investigating the sole effects of meteorological changes on reproduction as the periconceptional periods, patient’s physiological status, and the meteorological variables during the process of assisted reproduction can be easily and precisely determined [9]. Several studies have reported the seasonal variations in patients who underwent in vitro fertilization (IVF) treatment [9–14], but their results were controversial, although the studies were conducted under the same climatic conditions. In the UK, a study conducted in Bristol [10] found that seasonal variation is not associated with ovarian responsiveness or endometrial receptivity, whereas another study in Liverpool [11] found a higher implantation rate and pregnancy rate in summer cycles. A study from Israel [12] claimed that the IVF outcome fluctuations did not follow any specific seasonal pattern, whereas another study conducted in Jerusalem [9] found that the quality of the embryo and fertilization rates were affected by the season. The sub-arctic regions of Omsk, Russia, and Alberta, Canada share a similar humid continental climate, but the study in Omsk [13] showed a higher pregnancy rate in summer and autumn whereas the Alberta study [14] found no clear seasonal variation in the pregnancy rate.

The discrepancy in those studies may be due to the difference in the study population (from different age groups and/or stimulation protocols), difference in criteria for assigning a patient to the corresponding season (e.g., according to the day of stimulation, or the day of oocyte retrieval (OR) or the day of embryo transfer (ER)), and the use of an unsuitable method for the estimation of meteorological perturbation (only the average value for a month or a season was used).

Therefore, this study aimed to evaluate the effect of meteorological perturbation on the pregnancy rate in a cohort undergoing IVF treatment.

## Methods

### Study setting

This cohort study was conducted using the electronic IVF records from January 2005 to December 2016 of infertile patients who attended the Assisted Reproductive Unit, Prince of Wales Hospital of The Chinese University of Hong Kong, which is one of the largest public hospitals located in Hong Kong that has a humid sub-tropical climate with distinctive seasons based on the Koppen-Geiger classification system [15]. The cohort was divided into the following three groups: Group I included all eligible patients who underwent the first IVF cycle; Group II included patients with two cycles who had one successful and one failed pregnancy outcome as a case-control cohort for the intra-patient subgroup analysis; and Group III included all patients neglecting their number of treatment or cycle, and grouped their successful pregnancy events and variables in a monthly level to conduct an epidemic time-series study. The retrospective study was approved by the Joint Chinese University of Hong Kong – New Territories East Cluster Clinical Research Ethics Committee, and the requirement for obtaining written informed consent from the patients was waived.

### Patients’ eligibility criteria

The eligibility criteria were as follows: 1) age < 35 years to eliminate the effect of advanced age on fertility [16, 17]; 2) long protocol of ovarian stimulation cycle (The relatively long duration from drug administration to ER allowed the effect of meteorological variations to be manifested in the patients); and 3) fresh ER. Only stimulation followed by fresh ER can present the constant effect of meteorological variations.

The dates of gonadotropin administration (CYCL), OR, and ER were carefully examined. We traced the original medical records in patients with missing or incorrect electronic IVF records .

### Ovarian stimulation

During the period of study, the majority of the stimulation protocol used was the long protocol (860, 80%). Other stimulation protocols including the antagonist protocol were not included because there were too few cases for proper statistical analysis. Detailed information on the controlled ovarian hyperstimulation protocols is described in a previous literature [4]. In brief, pituitary down-regulation was achieved by the long luteal GnRH agonist (GnRH-a) down-regulation protocol. Buserelin nasal spray (Suprecur; Hoechst, Hørsholm, Germany) 1000 μg daily was given for at least 14 days from the mid-luteal phase of the preceding cycle. Complete pituitary desensitization was confirmed by low serum luteinizing hormone (LH) (< 10 IU/L) and estradiol (E2) (< 200 pmol/L) concentrations. Patients also had an ultrasound examination to exclude functional ovarian cysts and verify that endometrial thickness was < 5 mm. Once adequate down-regulation had been achieved, ovarian stimulation was started using hMG (Pergonal; Serono, Aubonne/Switzerland) or recombinant follicle-stimulating hormone (FSH) (Gonad-F; Serono; or Puregon; Organon, Skovlunde, Holland). The dose of gonadotropin was determined according to the age of the female partner and the previous responses to treatment if any.

### Embryo grading

Embryo grading criteria have been previously described [18]. Embryos were assessed based on the morphological criteria (number, size, and shape of blastomeres, the degree of fragmentation, the appearance of cytoplasm) and were categorized into five grades (grade 1 [worse] to grade 5 [best]). Embryos of ≥4 cells on Day 2 and ≥ 7 cells on Day 3 with < 10% fragmentation were regarded as good-quality embryos (grades 4 and 5). The top-quality embryos were chosen for transfer first, and if available, the blastocysts would be transferred before the cleavage stage embryos. If a patient had more than one embryo transferred, the grading was calculated as the highest of each embryo’s grading. Luteal phase support was provided either by progesterone administration or human chorionic gonadotropin (hCG) intramuscular injection from the day of OR. Luteal support was provided till 8–10 weeks of gestation.

### Pregnancy outcome measures

The outcomes of the IVF and ER cycles were assessed through serum hCG urine pregnancy test performed 10 days after the last dose of hCG luteal phase support or 2 weeks after ER, whichever came later. If it was positive, a vaginal ultrasound scan of the pelvis would be performed 2 weeks later to assess the site, the number, and the viability of gestation. The primary outcome measures of the study were PR (defined as a positive urine pregnancy test per cycle with ER). The live birth rate has not been included in this study because the gestation period far exceeds a single seasonal change and thus is unable to give a conclusive result.

### Meteorological and seasonal variations

Meteorological variations included were daily temperature, humidity, sunlight duration, and solar radiation from December 2004 to January 2017. These data were acquired from the Hong Kong Observatory, which is certified by the International Organization for Standardization ISO 9001:2015 Quality Management Systems for its public weather forecasting and warning services. These variations were matched with the duration of patients’ treatment daily — from the first day of CYCL to the day of ER, and the mean value of the matched variation over the CYCL-OR and OR-ER period was taken for analysis. We were also interested in seasonal variations, which present the overall effect of the long-term meteorological perturbation. Seasons were defined prior to data processing based on the Hong Kong Observatory definitions and a cycle was assigned to a season based on its ER day. Each season lasts for 3 months, as follows: Spring: March 1–May 31; Summer: June 1–August 31; Autumn: September 1–November 30; to Winter: December 1–February 28/29. Our IVF lab is certified to the ISO 9001:2008 Quality Management Standard for its design and provision of assisted reproductive technology treatments. The meteorological factors are stringently controlled in it. The room temperature is set to a range from 20 °C to 25 °C while the humidity is usually < 50%. The temperature perturbation of incubators and microscopes is limited at ±0.4 °C.

### Intra-patient subgroup analysis

To further confirm the meteorological effects and seasonal pattern showing significant effects on pregnancy outcome, the patients who underwent one successful cycle and one failed cycle were categorized into the part II cohort as a case-control cohort to eliminate all of the inter-patient variations as they were treated under the same protocol (long protocol). Mixed effects logistic regression models were developed for measuring the effect of different seasons and meteorology variables on the pregnancy probability.

### Time-series analysis

To further analyze the confirmed meteorological effects on pregnancy outcome at the epidemics level, we grouped all variables and successful pregnancy events in a month level and use distributed lag linear and non-linear models (DLNMs) to analyze their association.

### Statistical analysis

Continuous variables were described as medians and interquartile ranges (IQR) because all of them were not normally distributed. Categorical variables were described as proportions (%). The Kruskal-Wallis test was used to compare the mean of the numeric variables, whereas the chi-square test was used to compare the distribution of variables across different seasons.

We measure the effect of season and meteorology variables on the pregnancy probability in both individual and epidemic levels. For the individual level, we first selected the patients with the first treatment cycle and used logistic regressions to model the binary pregnancy outcome of each individual (successful or not). For each meteorology variable or season, univariate and multivariate models were built, respectively. Age, fertilization method, year of the cycle, gonadotropin dose, and transferred embryo grade were included in the multivariate model as covariates for each patient.

To further confirm the meteorological effects and seasonal pattern showing significance on pregnancy outcome, we analyzed a cohort of patients who underwent two cycles with only one clinical pregnancy outcome to eliminate all inter-patient variations. Given that these patients were treated under the same protocol (long protocol), each of their cycles can serve as a case-control cohort to reflect the true meteorological effects during the IVF treatment. We used the univariate and multivariate mixed-effect logistics regressions to control the repeated-measure structure of data and measure the effect of these variables. The covariates of the multivariate models were the same as mentioned previously, and the identity (ID) of each patient was set as a random effect to control the correlation between the same patients by giving different intercepts to each ID.

Besides, for meteorological effects showing significance, we analyzed their time-series trends in the epidemic level. To do this, the numeric variables were summarized to the month level by calculating the mean of each patient in a specific month, and categorical variables, such as fertilization method, were summarized as proportions. The DLNMs were used to evaluate the relationship between a meteorology variable and the number of successful pregnancy events separately [19]. Each model assumed an over-dispersed Poisson distribution of pregnancy events outcome and included a meteorology variable and covariates (mean age, year of the cycle, gonadotropin dose, transferred embryo grade, and proportion of fertilization method). We modelled the effect and lagged effect of meteorology variables through a natural cubic spline with 3 degrees of freedom and specified the lagged effect up to 6 months. The DLNM package was used to perform the modelling [20].

As for the patients aged < 35 years who received the antagonist protocol, we also conducted a descriptive analysis and individual level logistic regression to measure the association between meteorological effects and pregnancy outcome, since the results were non-significant (Additional file 1).

Results from the logistic regressions were presented as adjusted odds ratio (aOR) with 95% confidence interval (CI) and *P,* whereas those from DLNM were presented as relative risk (RR) with 95% CI. Results were considered significant if *P* < 0.05. Scatter plot with locally estimated scatterplot smoothing (LOESS) curves and 3D plot of RRs from DLNM was used to visualize the relationship between outcomes and variables. All analyses were performed using R 3.4.2.

## Results

### Patient characteristics

From 2005 to 2016, there were 860 patients with 1029 cycles who satisfied our inclusion criteria. In the 860 patients, all the first cycles of the patient were analyzed as Part I study, and 43 patients who had one successful cycle and one failed cycle were included in the part II intra-patient subgroup analysis. Demographic data of patients and laboratory outcomes are listed in Table 1. The median age of patients was 32 years, and the IQR was 31–34 years. The proportion of fertilization method, age, total gonadotropin dose, duration of controlled ovarian stimulation, LH level, E2 level, day 3 FSH level, and infertility diagnosis were balanced among the four seasons. The patients’ number of retrieved oocytes, fertilized oocytes, available embryos, and transferred embryo grades were balanced among the four seasons.

**Table 1:** Demographic information of patients and laboratory outcomes

Characteristics	Spring (*n* = 255)	Summer (*n* = 241)	Autumn (*n* = 194)	Winter (*n* = 170)	*P*
Fertilization method^a^					
IVF (n)	134	139	102	81	NS
Other (n)^c^	121	102	92	89	
Age (year)^b^	32(31–34)	32(31–34)	32(31–33)	32(31–34)	NS
Gn dose (IU), total^b^	2700(2250–3600)	2700(2250–3600)	2700(2250–3600)	2887.5(2250–3750)	NS
Stimulation period (day)^b^	11(10–12)	11(10–12)	11(10–12)	11(10–12)	NS
LH baseline^b, d^	1.9(1.2–3.0)	2.1(1.3–3.2)	1.8(1.2–2.8)	1.8(1.2–2.5)	NS
LH at trigger^b, d^	2.2(1.4–3.3)	2.2(1.5–3.5)	2.1(1.4–3.1)	1.8(1.3–2.7)	NS
E2 baseline^b, d^	53(44–77.8)	55(44–85)	49(44–71.8)	49(44–73.8)	NS
E2 at trigger^b, d^	12,706(8042–21,297)	11,556.5(7970.3–17,127.5)	11,316(7834.3–17,988.3)	10,576(6598.8–17,395)	NS
Day3 FSH^b, d^	6.9(5.9–8.1)	6.9(5.8–7.9)	6.8(5.8–7.9)	6.9(6.3–8)	NS
Infertility diagnosis^a^					
Tubal factor	133	132	107	85	NS
Pelvic Adhesions	110	110	90	62	NS
Uterine factor	5	2	5	2	NS
Male Factor	110	106	86	83	NS
Anovulation	33	32	24	20	NS
Endometriosis	47	48	34	26	NS
Sexual Dysfunction	3	2	2	6	NS
Immunological	1	0	1	0	NS
Unexplained	9	8	8	8	NS
Oocyte retrieved (n)^b^	11(8–14)	10(7–13)	10(7–14)	10(7–13)	NS
Fertilized oocyte (n)^b^	6(4–9)	6(4–8)	6(4–8)	5.5(4–8.75)	NS
Available embryos (n)^b, e^	3(2–6)	3(2–5)	3(2–5.8)	3(2–4)	NS
Transferred embryo grade^b, d, f^	4(4–4)	4(4–4)	4(4–4)	4(4–4)	NS

*NS* Non-significant

^a^*P* value was calculated using Chi-square test

^b^*P* value was calculated using Kruskal-Wallis test

^c^Other fertilization methods include ICSI, MESA, TESA

^d^Missing observations were removed (4 in LH baseline, 1 in E2 baseline, 7 in both LH and E2 at trigger and 38 in FSH, 4 in Transferred embryo grade)

^e^Available embryos are the sum of frozen and transferred embryos

^f^Transferred embryo grade of a cycle is the highest grade of all transferred embryo

### Influence of meteorological variations on pregnancy outcome

**Fig. 1:**
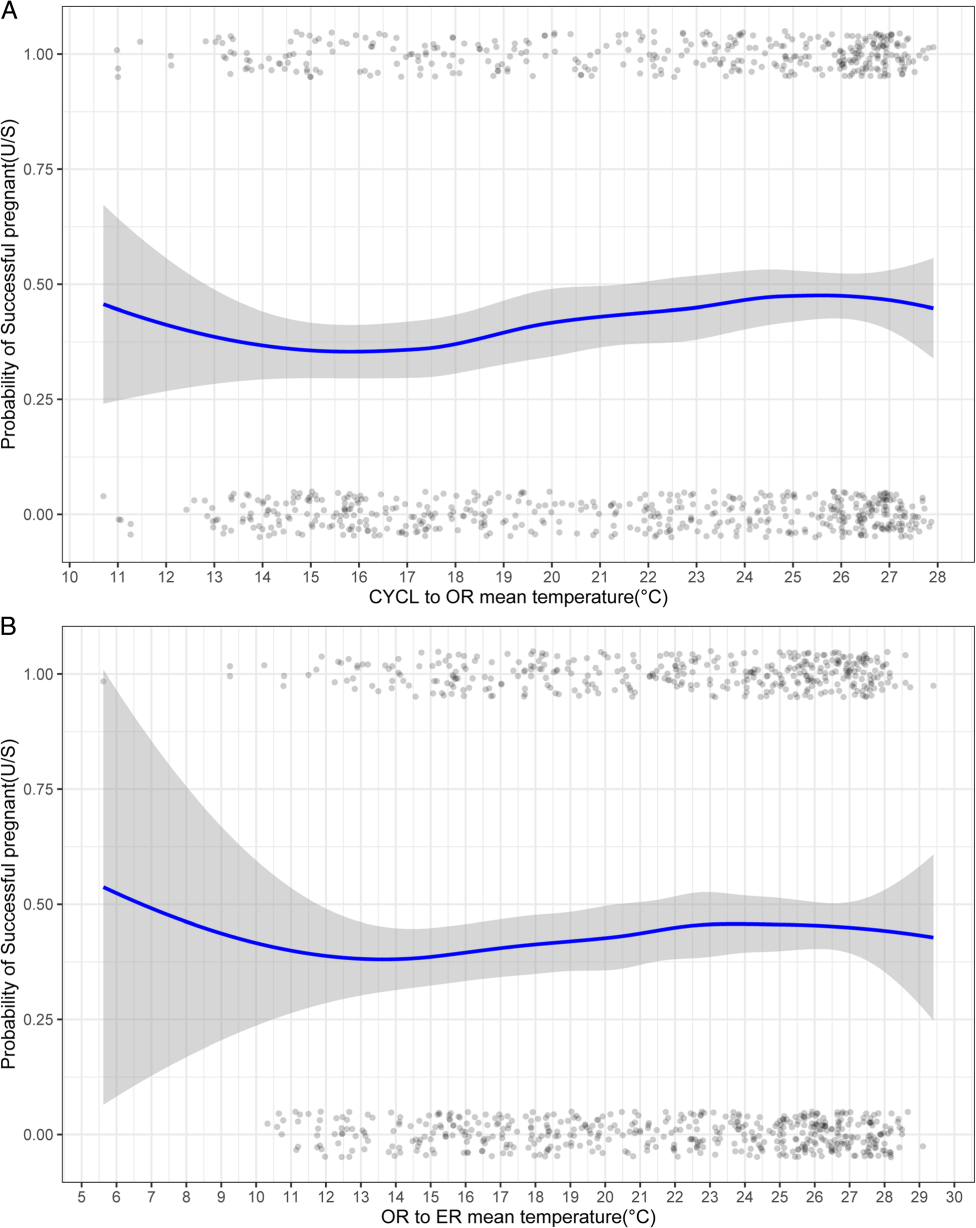
Association between the probability of pregnant and mean temperature. Upper (**a**) shows the duration from CYCL to OR while lower (**b**) shows the duration from OR to ER. Scatter plot reflects the number of events (pregnant or non-pregnant) as temperature changes. LOESS smoothing curve and 95% CI plot the relationship between the probability of pregnancy and month

The analysis of meteorological variation (Fig. 1a) shows that, overall, the pregnancy probability increased with temperature from the first day of drug administration to the day of ER. Table 2 shows the unadjusted (uOR) and adjusted odds ratio (aOR) and confidence interval (CI) range of the different meteorological variations from the univariate and multivariate analyses. Similar results can be seen in both analyses, and the multivariate analysis shows that the mean temperature from CYCL to OR have a significant association with pregnancy probability whereas that from OR to ER was non-significant. With each 1 °C increase in the mean temperature from CYCL to OR, the patients were 1.04 times more likely to become pregnant.

**Table 2:** Logistic regression analysis of the effect of meteorological variables on the pregnancy probability (pregnancy vs. non-pregnancy)

Variable	uOR (CI)	*P* for uOR	aOR (CI)	*P* for aOR
Mean temperature (°C)				
CYCL to OR	1.04(1.01–1.07)	0.01	1.04(1.01–1.07)	0.01
OR to ER	1.02(0.99–1.05)	NS	1.02(0.99–1.05)	NS
Mean humidity (%)				
CYCL to OR	1.01(0.99–1.04)	NS	1.01(0.99–1.04)	NS
OR to ER	1.00(0.98–1.01)	NS	1.00(0.98–1.01)	NS
Mean sunshine (h)				
CYCL to OR	1.04(0.95–1.14)	NS	1.05(0.95–1.15)	NS
OR to ER	1.00(0.95–1.05)	NS	0.99(0.95–1.05)	NS
Mean solar radiation (terajoules/square meter)				
CYCL to OR	1.03(0.98–1.07)	NS	1.02(0.98–1.06)	NS
OR to ER	1.01(0.99–1.04)	NS	1.01(0.98–1.04)	NS

*uOR* Unadjusted odds ratio

*aOR* Odds ratio after adjusting age, fertilization method, year of the cycle, gonadotropin dose and the grading of the transferred embryo

*CI* Confidence interval

*NS* Non-significant

### Influence of laboratory factors on pregnancy outcome

In the IVF treatment, in vitro embryo culture diverged from the time of OR and is one of the crucial parts that influence the pregnancy outcome. Therefore, we further analyzed whether the meteorological perturbation during embryo culture affected the pregnancy outcome. The duration from CYCL to ER was divided into two parts by the time point of OR — CYCL to OR and OR to ER. A brief timeline of the divergent point of OR, embryo culture and ER are shown in Additional file 2. The mean temperature from CYCL to OR still had a significant association with pregnancy probability, whereas OR to ER became non-significant (Table 2).

### Seasonal patterns on pregnancy outcome

**Fig. 2:**
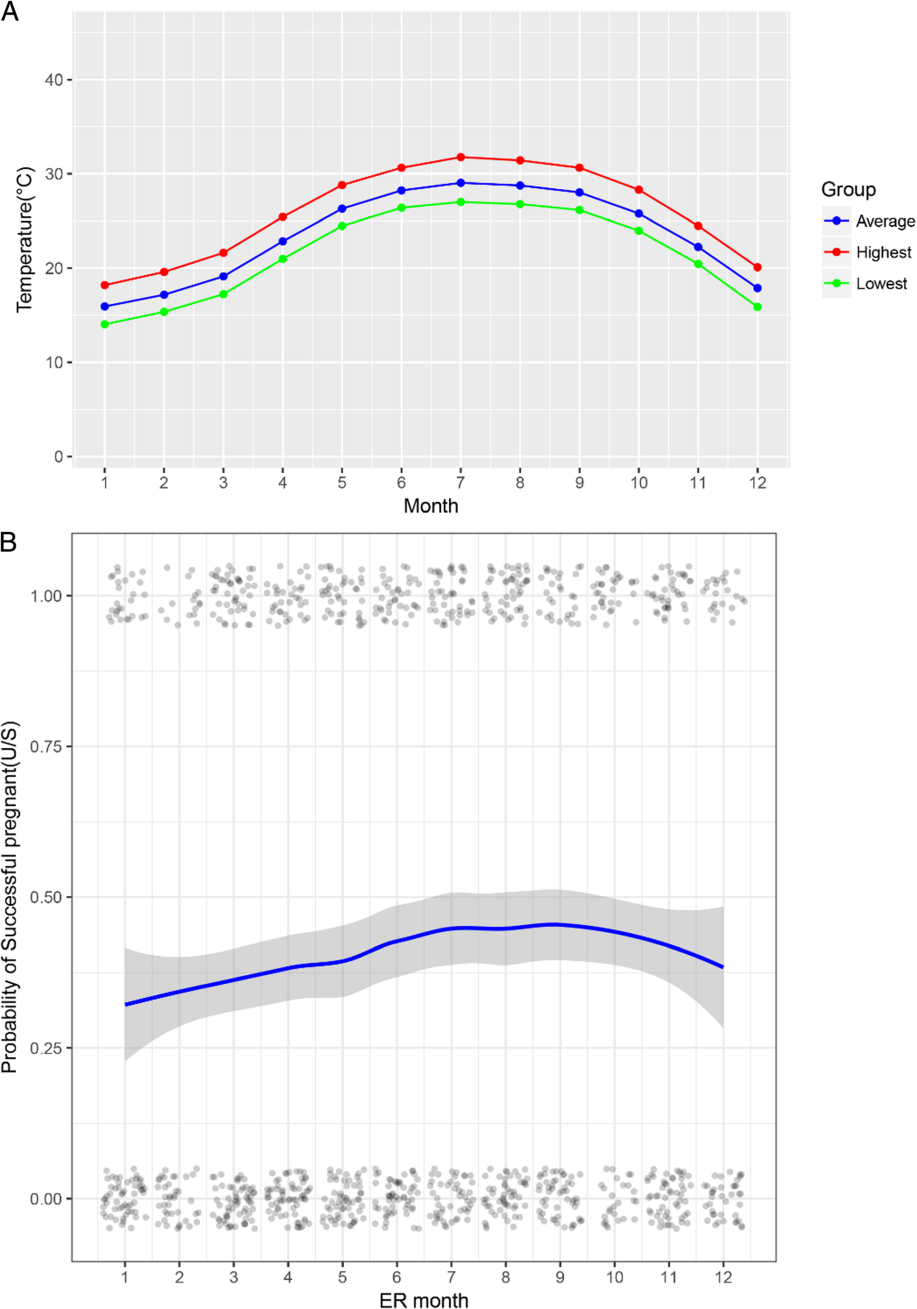
Upper (**a**): Monthly temperature combined from 2005 to 2016. The blue line there presents the combined average temperature in a specific month while the red line is for the average highest temperature and green line for the average lowest temperature. Lower (**b**): Association between the probability of becoming pregnant and ER month. Scatter plot reflects the number of events (pregnant or non-pregnant) in different months. LOESS smoothing curve and 95% CI plot the relationship between the probability of pregnancy and month

Our data showed that an increase in temperature has a positive effect on pregnancy outcome. Figure 2a shows a monthly temperature combined from 2005 to 2016, indicating that the seasons in Hong Kong are distinctive. Figure 2b shows the relationship between probability of pregnancy and ER month; more pregnancy events can be observed in July to September, and the smooth curve also shows a fast increasing trend followed by a decreasing trend, which is consistent with the aforementioned monthly temperature trend. Season variations have a significant association with pregnancy probability; compared with cycles in winter (December to February), the women with cycles in spring (March to May), summer (June to August), and autumn (September to November) were 1.238, 1.575, and 1.509 times more likely to become pregnant, respectively (Table 3).

**Table 3:** Logistic regression analysis of the effect of season on the pregnancy probability

Variable	uOR (CI)	*P* for uOR	aOR (CI)	*P* for aOR
Season				
Winter	Reference		Reference	
Spring	1.24(0.83–1.86)	NS	1.16(0.76–1.75)	NS
Summer	1.57(1.05–2.34)	0.03	1.47(0.97–2.23)	NS (0.07)
Autumn	1.80(1.18–2.74)	0.01	1.73(1.12–2.68)	0.02

*uOR* unadjusted odds ratio

*aOR* odds ratio after adjusting age, fertilization method, year of the cycle, gonadotropin dose and the grading of the transferred embryo

*CI* Confidence interval

*NS* Non-significant

### Intra-patient subgroup analysis

**Fig. 3:**
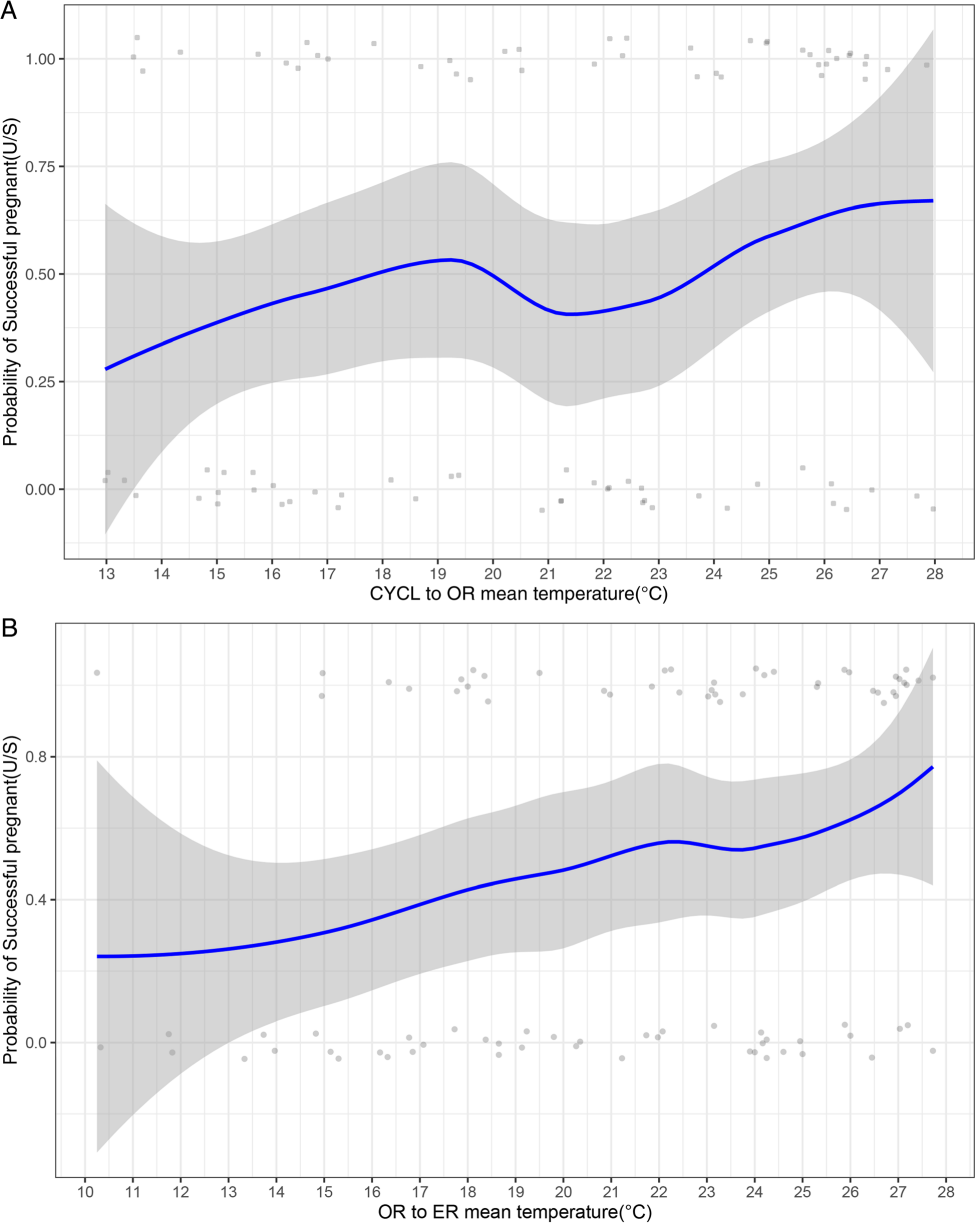
Association between the probability of pregnant and mean temperature in subgroup analysis. Upper (**a**) shows the duration from CYCL to OR while lower (**b**) shows the duration from OR to ER. Scatter plot reflects the number of events (pregnant or non-pregnant) as temperature changes. LOESS smoothing curve and 95% CI plot the relationship between the probability of pregnancy and month

The results of the intra-patient subgroup analysis are consistent with the one in the original population. Among our 1029 cycles, there were 43 patients who underwent two cycles with only one clinical pregnancy outcome. Demographic data of patients and laboratory outcomes are listed in Table 4. The distributions of both clinical and laboratory data of the subgroup population among four seasons were balanced. The analysis of meteorological variation (Fig. 3) shows, that in this subgroup of patients, the pregnancy probability was positively associated with the average temperature form CYCL to OR and OR to ER. Table 5 shows the unadjusted and adjusted OR and CI range of different meteorological variations. The multivariate analysis shows the mean temperature from CYCL to OR and OR to ER have a significant association with pregnancy probability (aOR = 1.12 and 1.14, respectively).

**Table 4:** Demographic information and laboratory outcomes of subgroup patients who underwent two cycles with only one clinical pregnancy outcome

Characteristics	Spring (*n* = 33)	Summer (*n* = 14)	Autumn (*n* = 22)	Winter (*n* = 17)	*P*
Fertilization method^a^					
IVF (n)	21	10	9	9	NS
Other (n)^c^	12	4	13	8	
Age (year)^b^	33(31–33)	32.5(31.3–34)	32(30–33)	32(31–33)	NS
Gn dose (IU), total^b^	2925(2475–3750)	2587.5(2300–3993.8)	3262.5(2700–4293.8)	3600(2475–4500)	NS
Stimulation period (day)^b^	11(10–12)	11(11–12)	11.5(11–12)	11(10–12)	NS
LH baseline^b^	2(1.3–3.1)	1.6(1.0–2.9)	1.3(0.9–3.0)	1.6(0.9–2.1)	NS
LH at trigger^b^	2(1.3–3.1)	2.0(0.9–3.1)	2.1(1.7–2.9)	1.6(1.2–1.9)	NS
E2 baseline^b,^	58(44–94)	57(44.25)	52(44–75.3)	48(44–54)	NS
E2 at trigger^b,^	11,242(7133–19,277)	13,713.5(8377–18,862.3)	12,054(7530–23,103)	9275(5772–12,038)	NS
Day3 FSH^b, d^	6.8(6.1–8.1)	7.5(6.8–8.2)	7.4(6.5–8.8)	7.4(6.3–8.4)	NS
Oocyte retrieved (n)^b^	9(8–14)	12(9–15.8)	9(6.3–11)	13(8–16)	NS
Fertilized oocyte (n)^b^	6(4–8)	5.5(3.3–9.5)	7(4–7)	6(5–9)	NS
Available embryos (n)^b, e^	2(2–4)	3(1.25–5)	2(2–3)	4(2–4)	NS
Transferred embryo^f^ grade^b^	4(4–4)	4(4–4)	4(4–4)	4(4–4)	NS

*NS* Non-significant

^a^*P* value was calculated using Chi-square test

^b^*P* value was calculated using Kruskal-Wallis test

^c^Other fertilization methods include ICSI, MESA, TESA

^d^Missing observations were removed (1 in LH baseline, 7 in FSH)

^e^Available embryos are the sum of frozen and transferred embryos

^f^Transferred embryo grade of a cycle is the highest grade of all transferred embryo

**Table 5:** Mixed effect logistic regression analysis of the effect of meteorological variables on the pregnancy probability (pregnancy vs. non-pregnancy)

Variable	uOR (CI)	*P* for uOR	aOR (CI)	*P* for aOR
Mean temperature (°C)				
CYCL to OR	1.10(1.00–1.21)	NS	1.12(1.01–1.24)	0.04
OR to ER	1.13(1.02–1.24)	0.02	1.14(1.03–1.26)	0.01
Season				
Winter	Reference		Reference	
Spring	3.45(0.93–12.83)	NS	4.07(1.01–16.44)	0.049
Summer	8.12(1.62–40.72)	0.01	11.63(1.95–69.35)	0.01
Autumn	3.90(0.96–15.82)	NS	5.29(1.18–23.64)	0.03

*uOR* Odds ratio only adjusting random effect of ID, without other covariate adjustment.

*aOR* Odds ratio after adjusting random effect of ID, age, fertilization method, year of the cycle, gonadotropin dose and the grading of the transferred embryo

*aOR* Adjusted Odds ratio

*CI* Confidence interval

*NS* Non-significant

**Fig. 4:**
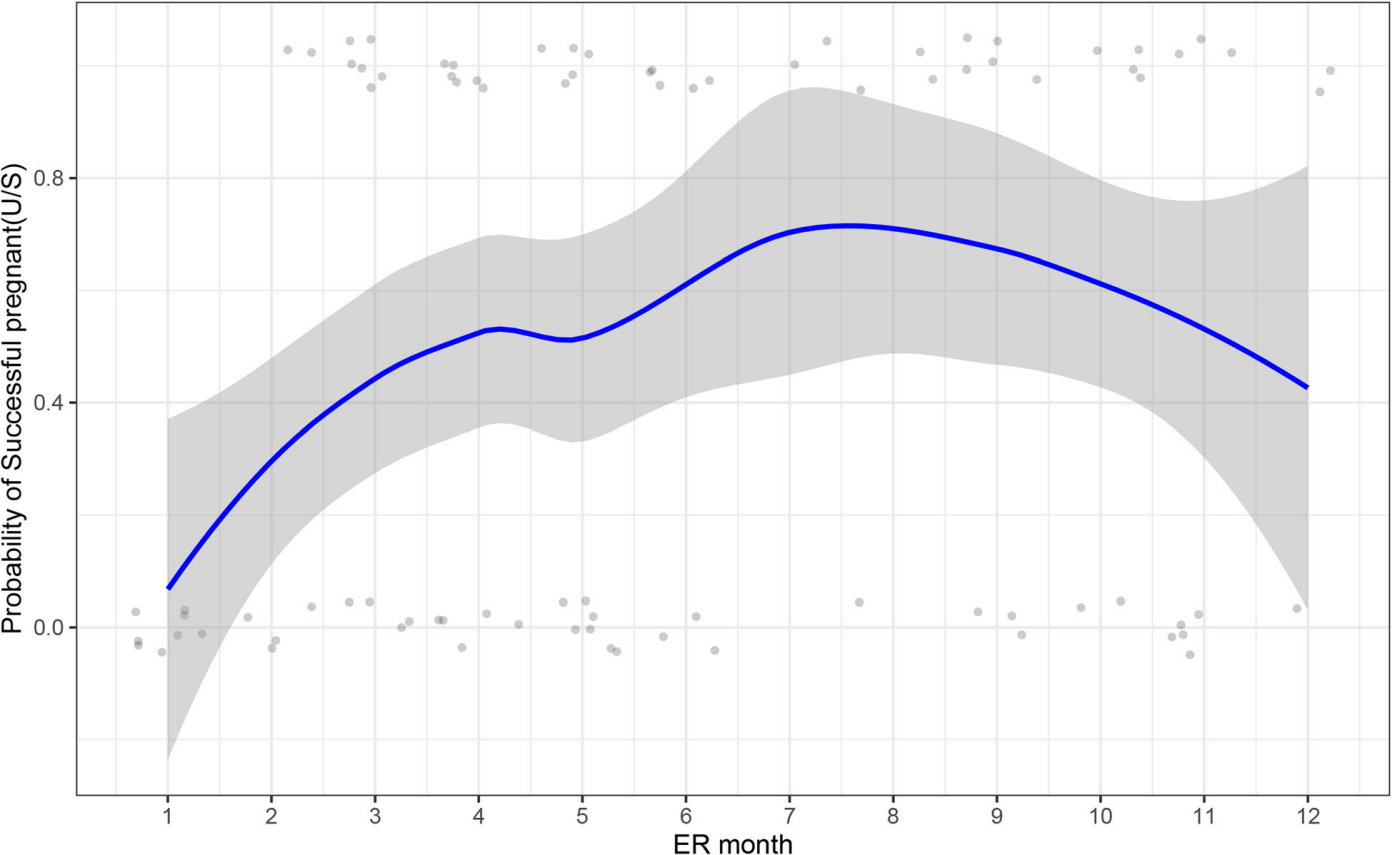
Association between the probability of becoming pregnant and ER month in subgroup analysis. Scatter plot reflects the number of events (pregnant or non-pregnant) in different months. LOESS smoothing curve and 95% CI plot the relationship between the probability of pregnancy and month

The relationship between probability of pregnancy and ER month in the subgroup analysis is shown in Fig. 4; more pregnancy events can be observed in the 7th–9th months (July to September), and the smooth curve also showed a fast increasing trend followed by a decreasing trend, which is consistent with the monthly temperature trend shown in Fig. 2a. Seasonal variations have a significant association with pregnancy probability; compared to that in winter, the patients were 4.07, 11.63 and 5.29 times more likely to become pregnant in spring, summer and autumn, respectively (Table 5).

### Epidemic time-series analysis

**Fig. 5:**
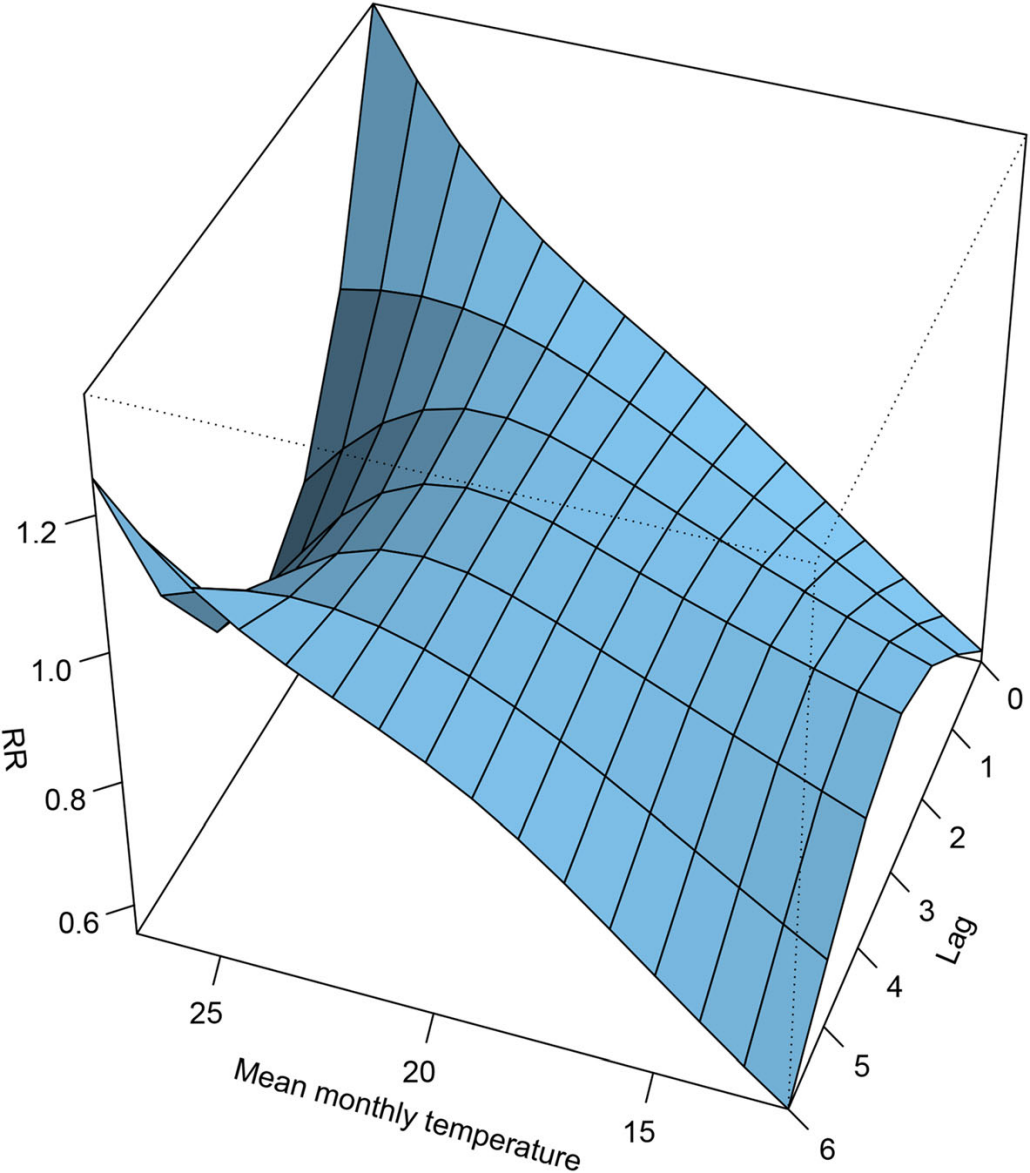
A comprehensive summary of the exposure-lag-response association. The x-axis, y-axis and z-axis represent the mean monthly temperature, lag of monthly mean temperature and RR, respectively. The reference value was the average temperature (21.5 °C)

The data of the original population was summarized with a monthly time-series format to explore the association between mean temperature and pregnancy events. The relationship between the effect of temperature and pregnancy events is shown in Fig. 5. A general positive association can be observed between temperature and the relative risk (RR) from 12 °C to 25 °C. A decreasing trend with fluctuation is then noted in different lags. The prediction of RR for different temperatures is shown in Table 6. With 21.5 °C as the reference temperature, the RR and its CI for each unit increase from 12 °C to 25 °C was calculated. The CI indicates that only the positive association between 12 °C and 16 °C was significant, and an increasing and decreasing trend is noted for the RR, with a turning point at 25 °C, which is similar to that shown in Fig. 5.

**Table 6:** Overall cumulative effect of temperature on pregnancy

Mean temperature (°C)	RR (CI)
12	0.07(0.01–0.6)
13	0.1(0.02–0.61)
14	0.14(0.03–0.66)
15	0.19(0.05–0.76)
16	0.26(0.08–0.91)
17	0.36(0.12–1.1)
18	0.47(0.18–1.25)
19	0.61(0.29–1.3)
20	0.77(0.48–1.22)
21	0.93(0.8–1.08)
21.5	Reference
22	1.07(0.92–1.26)
23	1.17(0.78–1.77)
24	1.21(0.66–2.21)
25	1.16(0.52–2.58)
26	1.04(0.33–3.29)
27	0.87(0.15–5.12)

*RR* Relative risk

## Discussion

Here, we evaluated the effects of meteorological variation on the pregnancy rate in a cohort undergoing IVF treatment and found that the changes in the ambient temperature markedly alters the pregnancy rates. To the best of our knowledge, this is the first study to evaluate the daily meteorological effects on pregnancy outcome in a sub-tropical region with the precise matching of meteorological data with the IVF stimulation cycle period. In this study, the patients’ clinical demographic data were nonsignificant for all seasons and for the number of retrieved oocytes, fertilized oocytes, and transferred embryo grade. Temperature is the most important factor affecting the pregnancy outcome among all the meteorological factors, whereas humidity, sunlight duration, and solar radiation had no effect on pregnancy outcome. Moreover, during seasons with higher average temperatures (summer and autumn), the pregnancy probability is higher than that during seasons with lower average temperature (spring and winter), indicating that higher temperature presents a dose-response gradient-like effect.

It should be noted that there are both similarities and differences between the first treatment and intra-patient subgroups. Although the positive association between average temperature and days from CYCL to OR were confirmed by both population, the effect of average temperature on days from OR to ER was significant in the intra-patient subgroup but not in the first treatment subgroup. Moreover, although the effect of autumn and summer on successful pregnancy was confirmed by both populations, the magnitude of these season measures by two models was different. This difference may be due to the small sample size of intra-patients as the CIs, which was wide, or due to the unadjusted and unmeasured covariates in the first treatment subgroup.

When we review the association in a time-series pattern and epidemic level, the analysis by the DNLM method provided another viewpoint for supporting the abovementioned findings. In lag 0–1, that is the month of treatment, the DNLM result shows trends consistent with the results of our regression analysis. Moreover, we found a significant effect—acute and long-term negative effect—of low temperature (< 16 °C) on pregnancy, which partially accounted for the relationship between temperature and pregnancy. However, although the trend of RR is clear, their CI for temperatures >16 °C has become non-significant. Thus, from the epidemic aspect, the relationship between temperature and pregnancy still need to be verified by further studies.

Besides, we tried to delineate whether the meteorological perturbation during the in vitro embryo culture is a confounding effect, by dividing the duration from the day of stimulation to the day of ER into in vivo and in vitro by the day of OR. Our data suggest that the association is only presented in the in vivo period (from stimulation cycle to OR) but not the in vitro period (from OR to ER)*;* implying the meteorological variations during embryo culture are not contributing to the association.

One of the largest studies on seasonal variation in IVF was conducted in Switzerland between 1995 and 2003, which included 7368 IVF cycles. They found that there were no statistically significant seasonal differences in Switzerland that influenced the IVF outcome. However, they admitted that the difference in stimulation protocols used attributed to the extent of discrepancies between seasonal variations studies in IVF [21]. In this cohort study, one of our inclusion criteria includes patients with a long ovarian protocol, which allows a longer period of downregulation compared to other protocols. In additional observation, we analysed the patient underwent antagonist protocol (Additional file 1, Section A), in which the meteorological variations did not show any significant effect. Moreover, when incorporated the antagonist protocol data into the long protocol data, the meteorological effect was masked (Additional file 1, Section B). This overall result may indicate that the meteorological effect was sensitive to stimulation protocol however the exact underlying reason was not well understood. Although the mechanism of temperature variations in human fertility is unknown, melatonin is one of the suspected affecting factors [22]. A study [6] on hamsters showed that female animals receiving melatonin during the afternoon became acyclic after several weeks of treatment and the acyclic female animals required 4–6 weeks to resume the estrous cyclicity following suspension of the melatonin injections. Therefore, a longer period may be beneficial for temperature variations to contribute to the metabolism of melatonin through the long-term ovarian protocol. Another explanation for the difference between the Switzerland study [21] and our study is the geographical difference. Switzerland covers a total surface area of >40,000 km^2^ with a spectrum of climates; thus, the average temperature measured at the meteorological stations may not truly reflect the temperature of patients in different areas; whereas Hong Kong just covers approximately 2754 km^2^ with multiple meteorological stations. Therefore, the parameter variants are more limited, and the data stringencies are higher.

Age is another important factor when analyzing the influence of meteorological variations. Women of advanced age tend to suffer from reduced fertility, especially in assisted reproduction treatment [17], and the decline in fertility and the increased time to conception occurs after the age of 35 [23]. To avoid this potential confounding variable on the pregnancy probability, the population in this study were limited to women aged < 35 years old.

For decades, the influence of seasonal variation on IVF has been extensively studied. However, the results remain controversial. Besides the factors of ovarian stimulation protocol and age as discussed above, the previous studies did not catch the essence of seasonal variation — temperature, humidity, sunlight duration, and solar radiation. For example, previous studies [21, 24, 25] analyzed the pregnancy outcome in different months ignoring the detailed meteorological perturbation in these durations. Although in the same season, the climates may vary from different years. Therefore, aligning daily meteorological variation data to the key duration of a patient’s treatment can precisely delineate in more detail their influence. This is the first report to use a daily meteorological parameter approach for analyzing their effects on pregnancy outcome. Among the four meteorological variations, temperature significantly influences pregnancy outcome, which is constant with our seasonal variation result — summer and autumn showed a higher likelihood of pregnancy probability.

The mechanism of the temperature influence on pregnancy probability is not yet completely understood, especially after we separately analyzed the duration from OR to ER when the gametes were manipulated in vitro. Similar results were presented by a study [13] conducted in the Siberian region with a temperate climate. They found that unsatisfactory IVF outcomes correlate with low air temperature and the best seasons for the IVF procedure are summer and autumn. However, its climate is characterized by marked changes in weather. In summer, the average daily temperature is + 20 °C, whereas in winter it decreases down to − 17 °C. Opposite results were presented by a study conducted in Taiwan [26], which is a region with tropical climate and higher average temperatures in the summer compared to those in Hong Kong and Siberia. The Taiwanese study investigated the correlation of the temperature, relative humidity, cumulative sunlight duration, and atmospheric pressure with IVF outcomes. They found that an increased temperature could have a negative association with pregnancy rate.

The Siberian and Taiwanese studies, along with this study, implicate that the temperature variations has an influence on IVF and the prospective outcome of pregnancy. However, due to the inconsistency of stimulation protocols and low accuracy of average temperature used in those studies, we hypothesize that there may be an optimal range for the temperature that can influence the pregnancy outcomes and no effect could be seen once the temperature is out of the range. On the other hand, the endometrium receptivity may be one of the contributing factors. A study in northern Europe showed that the increased incidence of endometrial hyperplasia was associated with low endometrial receptivity in the dark and long winter [27]. Temporary changes may influence the growth, development, and receptivity of the endometrium [2]. Nevertheless, in this study, the significant association between temperature and pregnancy outcome is observed, and we precisely aligned each day’s meteorological variations from the beginning of stimulation to the day of ER. This duration covered all the vital events of IVF procedures. This method also provides clinical evidence to resolve the problem that “there was no agreement between studies about the criterion for the inclusion of a patient to the corresponding season” addressed by Gindes et al. [12]. Moreover, this method allowed us to perform a more precise investigation of the impact of meteorological variations on each individual as well as on both in vivo and in vitro processes. The second advantage of this study is that we stratified our study population in the younger group as the effect of meteorological variations may be hidden if all ages are included in our dataset. Hong Kong is one of the most heavily populated cities, which has the highest population (7.5 million) density in < 2800 km^2^; thus, restricting environmental variation and diverse meteorological and geographical differences. The third advantage of this study is that we conducted an intra-patient subgroup analysis on the patients who underwent two cycles with at least one pregnancy outcome. The two cycles of these patients served as self-control for eliminating individual variations and further confirmed the findings in the larger population of our 1029 cycles.

Although we have attempted to minimize the study limitations, there are still some limitations of this study. The meteorological variations were measured outdoors, and most places in Hong Kong have air-conditioning indoors, which affects the temperature and humidity. However, all patients would inevitably be affected by the outdoor environment, irrespective of the duration of indoor activities. Another limiting factor is that the meteorological effect before the IVF cycle cannot be evaluated. However, our results show that a long stimulation protocol is favorable in significantly increasing the pregnancy rate in summer and autumn. Lastly, the study population is of a specific age and underwent a specific stimulation protocol; this should be considered when applying our conclusions to other subgroup populations.

## Conclusions

The results of our highly stringent approach, wherein daily meteorological variations were aligned with the specific subgroup of patients in the sub-tropical region, and the subgroup analysis showed that the increased average temperature in summer and autumn has a positive impact on the pregnancy rate, an aspect that should be considered when obtaining patients’ consent for assisted conception. Studies investigating the underlying physiological mechanism acting in response to environmental cues are warranted.

## Supplementary information

**Additional file 1.**

**Additional file 2.**

## Data Availability

Please contact the author for data requests.

## Abbreviations

ANOVA: Analysis of variance
aOR: Adjusted odds ratio
ART: Assisted reproductive technology
CI: Confidence interval
CYCL: The date of gonadotropin administration
E2: Estradiol
ER: Embryo transfer
FSH: Follicle-stimulating hormone
GnRH-a: Gonadotropin-releasing hormone agonist
hCG: Human chorionic gonadotropin
hMG: Human menopausal gonadotropin
IVF: In-vitro fertilization
LH: Luteinizing hormone
LOESS: Locally estimated scatterplot smoothing
OR: Oocyte retrieval
SD: Standard deviation
