# Supplementary figures and images for: The effects of daily meteorological perturbation on pregnancy outcome: follow-up of a cohort of young women undergoing IVF treatment
Source: Environ Health. 2019 Nov 28;18:103. doi: 10.1186/s12940-019-0538-7 (PMC6883622; doi:10.1186/s12940-019-0538-7)

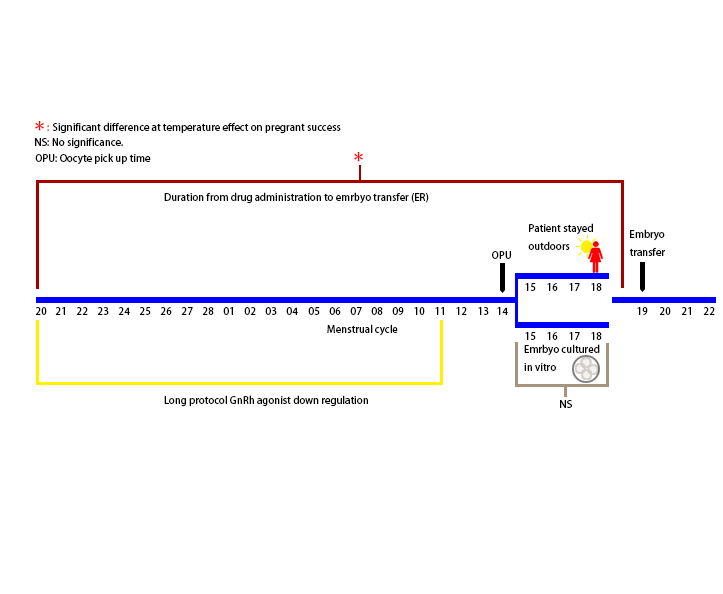

Supplement: Supplementary file 1 — Additional file 1. [file 12940_2019_538_MOESM1_ESM.tif]
