# Supplementary material for: The effects of daily meteorological perturbation on pregnancy outcome: follow-up of a cohort of young women undergoing IVF treatment
Source: Environ Health. 2019 Nov 28;18:103. doi: 10.1186/s12940-019-0538-7 (PMC6883622; doi:10.1186/s12940-019-0538-7)
Supplement: Supplementary file 2 — Additional file 2. [file 12940_2019_538_MOESM2_ESM.docx]

**Section A**

Analysis of first treatment patients under 35-year old and experienced the antagonist protocol

The characteristics of patients was listed in Table SA_1. The results of logistic regression for the association between meteorological effects and pregnancy outcome were listed in Table SA_2 and Table SA_3. In this population, all demographic variables were balanced among 4 seasons and the effect of meteorological variables were non-significant. Multiple logistic regressions adjusted for age, fertilization method, year of the cycle, gonadotropin dose and the grading of the transferred embryo.

**Table SA_1. Demographic information and laboratory outcomes of patients underwent antagonist protocol.**

| Characteristics | | Spring (n=50) | Summer (n=56) | Autumn (n=75) | Winter (n=76) | P |
| --- | --- | --- | --- | --- | --- | --- |
| Fertilization method ^a^ | IVF (n) | 27 | 32 | 25 | 21 | NS |
|  | Other (n)^c^ | 48 | 44 | 31 | 29 |  |
| Age (year)^b^ | | 32(31-33.5) | 33(32-34) | 33(31-34) | 32(30-33) | NS |
| Gn dose (IU), total ^b^ | | 3000(2175-4500) | 2962.5(2175-4500) | 3187.5(2025-4275) | 2325(2043.8-3750) | NS |
| Stimulation period (day)^b^ | | 10(9-12) | 10(9-13) | 10(9-11) | 10(9.25-11) | NS |
| LH baseline ^b, d^ | | 4.6(2.6-6.1) | 5.2(3.7-6.5) | 5.0(3.4-6.6) | 4.1(3-5.5) | NS |
| LH at trigger ^b, d^ | | 2.2(1.3-3.9) | 2.4(1.4-3.2) | 2.1(1.2-3.2) | 2.1(1.4-2.7) | NS |
| E2 baseline ^b, d^ | | 98(55.8-132.5) | 88.5(48.8-141.3) | 85(61-141.3) | 91(67-128) | NS |
| E2 at trigger ^b, d^ | | 7390(5078-9579.5) | 8108(5366-10829.8) | 6838(5582-11009) | 8787(6936-13682.5) | NS |
| Day3 FSH ^b, d^ | | 7.6(6.3-9.1) | 7.4(6.4-9.1) | 7.9(5.9-9.9) | 7.2(6.1-8.3) | NS |
| Infertility diagnosis ^a^ | Tubal factor | 32 | 27 | 24 | 23 | NS |
|  | Pelvic Adhesions | 22 | 26 | 15 | 19 | NS |
|  | Uterine factor | 0 | 0 | 0 | 0 | NS |
|  | Male Factor | 42 | 39 | 32 | 25 | NS |
|  | Anovulation | 14 | 15 | 9 | 10 | NS |
|  | Endometriosis | 14 | 15 | 7 | 8 | NS |
|  | Sexual Dysfunction | 0 | 2 | 2 | 2 | NS |
|  | Immunological | 0 | 0 | 0 | 0 | NS |
|  | Unexplained | 0 | 0 | 1 | 2 | NS |
| Oocyte retrieved (n) ^b^ | | 8(5.5-11) | 8(7-11.25) | 9(6-13.25) | 9.5(7-14) | NS |
| Fertilized oocyte (n)^b^ | | 5(3-8) | 6(3.8-8) | 6(3-9) | 6(4-8.8) | NS |
| Available embryos (n)^b, e^ | | 2(2-4.5) | 2(2-4) | 3(2-4) | 3(2-4) | NS |
| Transferred embryo grade ^b, f^ | | 4(4-4) | 4(4-4) | 4(4-4) | 4(3-4) | NS |
|  | |  |  |  |  |  |

^a^ P value was calculated using Chi-square test;

^b^ P value was calculated using Kruskal-Wallis test;

^c^ Other fertilization methods include ICSI, MESA, TESA;

^d^ Missing observations were removed (1 in both LH and E2 baseline, 3 in both LH and E2 at trigger and 22 in FSH, 5 in Transferred embryo grade);

^e^ Available embryos are the sum of frozen and transferred embryos;

^f^ Transferred embryo grade of a cycle is the highest grade of all transferred embryo.

NS = non-significant

**Table SA_2. Logistic regression analysis of the effect of meteorological variables on the pregnancy probability (pregnancy vs. non-pregnancy) in patients underwent antagonist protocol.**

| Variable | | OR(CI) | P for OR | aOR (CI) | P for aOR | | |
| --- | --- | --- | --- | --- | --- | --- | --- |
| Mean temperature (℃) | CYCL to ER | 0.97(0.92-1.03) | NS | 0.98(0.92-1.03) | | NS |  |
|  | CYCL to OR | 0.98(0.93-1.03) | NS | 0.98(0.92-1.03) | | NS |  |
|  | OR to ER | 0.98(0.93-1.03) | NS | 0.98(0.92-1.03) | | NS |  |
|  |  |  |  |  | |  |  |
| Mean humidity (%) | CYCL to ER | 0.99(0.95-1.03) | NS | 0.99(0.95-1.03) | | NS |  |
|  | CYCL to OR | 0.99(0.96-1.02) | NS | 0.99(0.96-1.03) | | NS |  |
|  | OR to ER | 0.99(0.96-1.02) | NS | 0.99(0.96-1.03) | | NS |  |
|  |  |  |  |  | |  |  |
| Mean sunshine (h) | CYCL to ER | 1.00(0.86-1.17) | NS | 1.01(0.86-1.19) | | NS |  |
|  | CYCL to OR | 1.03(0.90-1.19) | NS | 1.04(0.90-1.21) | | NS |  |
|  | OR to ER | 0.97(0.88-1.08) | NS | 0.99(0.89-1.10) | | NS |  |
| Mean solar radiation (terajoules/square meter) |  |  |  |  | |  |  |
|  | CYCL to ER | 0.99(0.92-1.06) | NS | 0.99(0.92-1.07) | | NS |  |
|  | CYCL to OR | 1.00(0.93-1.07) | NS | 1.00(0.93-1.08) | | NS |  |
|  | OR to ER | 0.98(0.93-1.19) | NS | 0.99(0.93-1.05) | | NS |  |

OR = odds ratio without covariate adjustment

aOR = odds ratio after adjusting age, fertilization method, year of the cycle, gonadotropin dose and the grading of the transferred embryo

CI = confidence interval

NS = non-significant

**Table SA_3. Logistic regression analysis of the effect of season on the pregnancy probability patients underwent antagonist protocol.**

| Variable |  | OR(CI) | P for OR | aOR (CI) | P for aOR |
| --- | --- | --- | --- | --- | --- |
| Season | Winter | Reference |  | Reference |  |
|  | Spring | 0.68(0.32-1.41) | NS | 0.74(0.35-1.58) | NS |
|  | Summer | 0.74(0.36-1.54) | NS | 0.78(0.36-1.69) | NS |
|  | Autumn | 0.47(0.21-1.05) | NS | 0.46(0.19-1.08) | NS |

OR = odds ratio without covariate adjustment

aOR = odds ratio after adjusting age, fertilization method, year of the cycle, gonadotropin dose and the grading of the transferred embryo

CI = confidence interval

NS = non-significant

**Section B**

Analysis of first treatment patients under 35-year old and experienced the antagonist or long protocol

The characteristics of patients was listed in Table SB_1. The results of logistic regression for the association between meteorological effects and pregnancy outcome were listed in Table SB_2 and Table SB_3. In this population, all demographic variables were balanced among 4 seasons and the effect of meteorological variables were non-significant. Multiple logistic regressions adjusted for age, fertilization method, year of the cycle, gonadotropin dose and the grading of the transferred embryo.

Note that the number of first treatment patients experienced the antagonist or long protocol are less than the summation of first treatment patients experienced the antagonist protocol and that experienced the long protocol because some patients experienced both long and antagonist protocol but count as 1 record in this dataset.

**Table SB_1. Demographic information and laboratory outcomes of patients underwent long or antagonist protocol.**

| Characteristics | | Spring (n=318) | Summer (n=305) | Autumn (n=247) | Winter (n=215) | P |
| --- | --- | --- | --- | --- | --- | --- |
| Fertilization method ^a^ | IVF (n) | 157 | 166 | 125 | 100 | NS |
|  | Other (n)^c^ | 161 | 139 | 122 | 115 |  |
| Age (year)^b^ | | 32(31-34) | 32(31-34) | 32(31-33) | 32(30-33.5) | NS |
| Gn dose (IU), total ^b^ | | 2700(2250-3600) | 2700(2250-3600) | 2700(2250-3675) | 2700(2250-3750) | NS |
| Stimulation period (day)^b^ | | 11(10-12) | 11(10-12) | 11(10-12) | 11(10-12) | NS |
| LH baseline ^b, d^ | | 2.2(1.3-3.6) | 2.4(1.4-3.9) | 2.1(1.3-3.5) | 2.1(1.3-3.3) | NS |
| LH at trigger ^b, d^ | | 2.2(1.4-3.6) | 2.3(1.5-3.4) | 2.1(1.4-3.1) | 1.9(1.3-2.7) | NS |
| E2 baseline ^b, d^ | | 57.5(44-91.3) | 59(44-93) | 58(44-83) | 55(44-88) | NS |
| E2 at trigger ^b, d^ | | 10939(7214.3-19403) | 10775(7363-15272) | 10227(6939-15660) | 10281(6612-16220) | NS |
| Day3 FSH ^b, d^ | | 7(6-8.3) | 7.1(6.1-8) | 6.9(5.8-8.4) | 7(6.2-8) | NS |
| Infertility diagnosis ^a^ | Tubal factor | 159 | 153 | 129 | 105 | NS |
|  | Pelvic Adhesions | 126 | 129 | 103 | 79 | NS |
|  | Uterine factor | 5 | 11 | 5 | 3 | NS |
|  | Male Factor | 147 | 141 | 117 | 105 | NS |
|  | Anovulation | 44 | 45 | 33 | 29 | NS |
|  | Endometriosis | 60 | 59 | 41 | 33 | NS |
|  | Sexual Dysfunction | 3 | 4 | 4 | 8 | NS |
|  | Immunological | 1 | 0 | 1 | 0 | NS |
|  | Unexplained | 15 | 13 | 10 | 10 | NS |
| Oocyte retrieved (n) ^b^ | | 10(7-13.75) | 10(7-13) | 10(7-14) | 10(7-13) | NS |
| Fertilized oocyte (n)^b^ | | 6(4-9) | 6(4-8) | 6(4-8) | 6(4-8.5) | NS |
| Available embryos (n)^b, e^ | | 3(2-5) | 3(2-5) | 3(2-5) | 3(2-4) | NS |
| Transferred embryo grade ^b, f^ | | 4(4-4) | 4(4-4) | 4(4-4) | 4(3-4) | NS |
|  | |  |  |  |  |  |

^a^ P value was calculated using Chi-square test;

^b^ P value was calculated using Kruskal-Wallis test;

^c^ Other fertilization methods include ICSI, MESA, TESA;

^d^ Missing observations were removed (5 in LH baseline, 8 in both LH and E2 at trigger, 2 in E2 baseline, and 57 in FSH, 9 in Transferred embryo grade);

^e^ Available embryos are the sum of frozen and transferred embryos;

^f^ Transferred embryo grade of a cycle is the highest grade of all transferred embryo.

NS = non-significant

**Table SB_2. Logistic regression analysis of the effect of meteorological variables on the pregnancy probability (pregnancy vs. non-pregnancy) in patients underwent long or antagonist protocol.**

| Variable | | OR(CI) | P for OR | aOR (CI) | P for aOR | | |
| --- | --- | --- | --- | --- | --- | --- | --- |
| Mean temperature (℃) | CYCL to ER | 1.02(1.00-1.05) | NS (0.06) | 1.02(1.00-1.05) | | NS (0.08) |  |
|  | CYCL to OR | 1.03(1.00-1.05) | NS (0.05) | 1.02(1.00-1.05) | | NS (0.07) |  |
|  | OR to ER | 1.01(0.99-1.04) | NS (0.06) | 1.01(0.99-1.04) | | NS (0.08) |  |
|  |  |  |  |  | |  |  |
| Mean humidity (%) | CYCL to ER | 1.01(0.99-1.03) | NS | 1.00(0.98-1.02) | | NS |  |
|  | CYCL to OR | 1.01(0.99-1.03) | NS | 1.00(0.98-1.02) | | NS |  |
|  | OR to ER | 1.00(0.98-1.01) | NS | 0.99(0.98-1.01) | | NS |  |
|  |  |  |  |  | |  |  |
| Mean sunshine (h) | CYCL to ER | 1.03(0.95-1.11) | NS | 1.03(0.95-1.12) | | NS |  |
|  | CYCL to OR | 1.04(0.96-1.12) | NS | 1.05(0.97-1.13) | | NS |  |
|  | OR to ER | 1.00(0.96-1.04) | NS | 1.00(0.95-1.04) | | NS |  |
| Mean solar radiation (terajoules/square meter) |  |  |  |  | |  |  |
|  | CYCL to ER | 1.02(0.98-1.06) | NS | 1.02(0.98-1.06) | | NS |  |
|  | CYCL to OR | 1.02(0.98-1.06) | NS | 1.02(0.98-1.06) | | NS |  |
|  | OR to ER | 1.01(0.98-1.03) | NS | 1.01(0.98-1.03) | | NS |  |

OR = odds ratio without covariate adjustment

aOR = odds ratio after adjusting age, fertilization method, year of the cycle, gonadotropin dose and the grading of the transferred embryo

CI = confidence interval

NS = non-significant

**Table SB_3. Logistic regression analysis of the effect of season on the pregnancy probability in patients underwent long or antagonist protocol.**

| Variable |  | OR(CI) | P for OR | aOR (CI) | P for aOR |
| --- | --- | --- | --- | --- | --- |
| Season | Winter | Reference |  | Reference |  |
|  | Spring | 1.12(0.79-1.60) | NS | 1.06(0.74-1.53) | NS |
|  | Summer | 1.38(0.96-1.97) | NS（0.08） | 1.30(0.91-1.88) | NS |
|  | Autumn | 1.33(0.92-1.94) | NS | 1.29(0.88-1.89) | NS |

OR = odds ratio without covariate adjustment

aOR = odds ratio after adjusting age, fertilization method, year of the cycle, gonadotropin dose and the grading of the transferred embryo

CI = confidence interval

NS = non-significant
